# SEAS: A System for SEED-Based Pathway Enrichment Analysis

**DOI:** 10.1371/journal.pone.0022556

**Published:** 2011-07-22

**Authors:** Xizeng Mao, Yu Zhang, Ying Xu

**Affiliations:** 1 Computational Systems Biology Lab, Department of Biochemistry and Molecular Biology, Institute of Bioinformatics, University of Georgia, Athens, Georgia, United States of America; 2 BioEnergy Science Center BESC, University of Georgia, Athens, Georgia, United States of America; 3 College of Computer Science and Technology, Jilin University, Changchun, China; 4 Key Laboratory of Symbolic Computation and Knowledge Engineering of the Ministry of Education, Jilin University, Changchun, China; Dana-Farber Cancer Institute, United States of America

## Abstract

Pathway enrichment analysis represents a key technique for analyzing high-throughput *omic* data, and it can help to link individual genes or proteins found to be differentially expressed under specific conditions to well-understood biological pathways. We present here a computational tool, SEAS, for pathway enrichment analysis over a given set of genes in a specified organism against the pathways (or subsystems) in the SEED database, a popular pathway database for bacteria. SEAS maps a given set of genes of a bacterium to pathway genes covered by SEED through gene ID and/or orthology mapping, and then calculates the statistical significance of the enrichment of each relevant SEED pathway by the mapped genes. Our evaluation of SEAS indicates that the program provides highly reliable pathway mapping results and identifies more organism-specific pathways than similar existing programs. SEAS is publicly released under the GPL license agreement and freely available at http://csbl.bmb.uga.edu/~xizeng/research/seas/.

## Introduction

High-throughput *omic* techniques are being increasingly more widely used by large research centers as well as by individual labs because of the rapidly decreasing costs and the increasing quality of the data generated. The rapid accumulation of the *omic* data has provided unprecedented new opportunities for biologists to study substantially more complex problems at a systems level [1], [2] than just a few years ago. As a key technique in linking individual genes/proteins to biological processes, pathway enrichment analysis is being widely used to study pathway-level activities based on the activities of individual genes/proteins observed using *omic* techniques [3], [4]. A number of computational tools have been developed to provide pathway enrichment analyses against different pathway databases. As of now, the majority of the existing tools have been designed for pathway analyses for human or eukaryotes in general, including ArrayXPath [5], GenMAPP [6], DAVID [7], PathwayExplorer [8], PathExpress [9] and Pathway Miner [10]. Among all these analysis tools, gene mapping from a specified organism to the pathway genes covered by the underlying (pathway) database is typically done through gene ID [5], [6], [7] or orthology mapping [11], [12]. A pathway is considered as enriched by a set of genes if they overlap the pathway at a substantially higher percentage of the pathway genes than expected by chance. Statistical enrichment analysis methods fall into three classes according to enrichment algorithms [13]: (i) singular enrichment analysis (SEA), which calculates an enrichment *P-value* on each pathway and lists the enriched pathways in a linear table based on the hyper-geometric distribution assumption [14] or using Fisher exact test [15], [16] among a few other methods [17] [18]; (ii) gene set enrichment analysis [19], which considers an entire gene set (without pre-selection) encoded in a genome and associated experimental values (for instance expression fold change); and (iii) modular enrichment analysis [20], which uses the key idea of SEA but considers pathway-pathway or gene-gene relations in its enrichment P-value calculation. In this paper, we will use the SEA method because of its simplicity and popularity, and may consider the other two classes of enrichment analysis methods in our future work.

Currently there are a few popular pathway databases in the public domain, without a particular one being the predominant one [21], as they each have their own strengths and limitations, making each of them suitable for different application scenarios. For example, the KEGG Pathway database [22] has a collection of generic pathways mostly derived based on known biochemical reactions rather than how individual organisms execute the reactions. Hence these generic pathways could be considered as a superset of the corresponding pathways specific to individual organisms, i.e., not every reaction in a KEGG pathway is encoded in every organism [23]. So mapping these generic pathways to specific organisms generally requires manual examination to ensure the mapping quality. The SEED Subsystem database is another pathway resource; each subsystem (pathway) for a specific organism in SEED is constructed by a group of domain experts [24], making its pathway genes more organism-specific and generally more reliable than KEGG pathways. Its limitation is that its coverage might not be as high as KEGG pathways. For example, the KEGG pathways cover 2,983 *E. coli* genes while SEED covers only 2,181 while exceptions exist. For instance, KEGG covers 2,296 *B. subtilis* genes while SEED covers 2,303.

We have previously developed a software tool KOBAS [11] for enrichment analyses of KEGG pathways, which has been widely used since its publication [25]. Here we present a new tool for enrichment analyses against SEED subsystems, called SEAS (**S**EED-based **E**nrichment **A**nalysis **S**ystem). SEAS provides three ways for gene mapping to subsystems through gene ID, orthology or homology mapping based on the availability of the relevant information, and identifies the statistically enriched pathways in SEED. We have extensively tested the performance of SEAS by re-annotating known pathways of *E. coli* and *B. subtilis* in SEED, and found that the mapped pathways are highly reliable, achieving 79% precision and 95% coverage for *E. coli* and 66% precision and 74% coverage for *B. subtilis*. Our additional evaluation results on microarray data and newly sequenced genome suggest that SEAS can identify more organism-specific pathways than KEGG-based pathway annotation. To the best of our knowledge, SEAS is the first software for SEED pathway enrichment analysis.

## Results and Discussion

**Figure 1:**
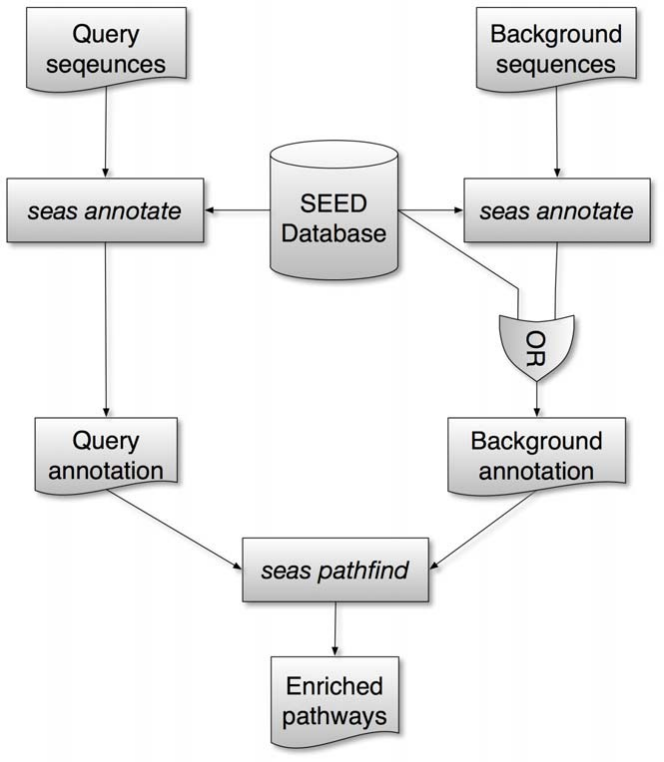
A schematic representation of the SEAS workflow. Each rectangle represents a program, each cylinder represents a database, and the others are flat text files for input, output or intermediate results.

The workflow of SEAS consists of two main steps as shown in Figure 1: (a) it first maps the query genes to SEED subsystems based on sequence similarity search or ID mapping; and (b) it then compares the ratio of the query genes out of all the genes in each mapped subsystem *versus* the ratio of the query genes out of the whole gene set of the query genome or some other background ratio prepared by the user, and identifies significantly enriched subsystems.

### Gene mapping to pathways by multiple strategies

Mapping the query genes to pathways involves searching the well-annotated gene database in SEED that currently has 1,414 organisms. We have implemented three strategies in SEAS, one of which will be used depending on the availability of the relevant information. When the query genes are already in SEED, we will use the original (pathway) annotation in SEED directly if the SEED ID is available for the query or through ID mapping using the NCBI GI number as the universal ID. When the genes are not covered but have available genome in SEED, we will use the mapping results between the query genes and the pathway genes in SEED given by the official RAST server using Bi-Directional Best Hit (BDBH) [26], or use the mapping results by our own P-MAP program [27] when operons for the query genome are available. P-MAP uses both high sequence similarity and operon information for orthologous gene mapping, and hence tend to make the mapping results more accurate than BDBH when it is applicable. When neither of these two methods provides useful mapping results, which could be true for partially sequenced genomes and meta-genomes, we use NCBI BLAST (*blastp* for DNA, *blastx* for protein) (see Material and Methods on E-value cutoff), to compare the query genes/proteins against one or more reference genomes in SEED specified by the user, in which we select the top hit with known annotation in SEED. The SEAS program provides the option for the user to choose one of the options to do gene mapping.

**Figure 2:**
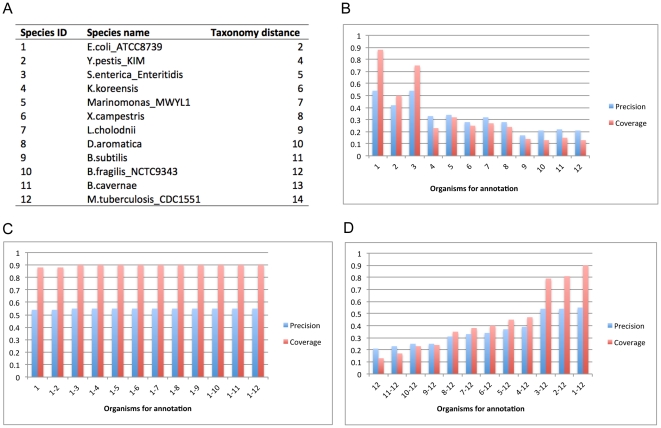
SEAS-based re-annotation of *E. coli* pathways using 11 reference genomes. (A) Taxonomic distance between reference genomes and *E. coli*. The first column represents the reference genomes, used in the *x*-axis in (B)–(D); (B) Re-annotation of *E. coli* pathways using the *single genome strategy*; (C) Re-annotation of *E. coli* pathways using the *multiple genome strategy #1*; (D) Re-annotation of *E. coli* pathways using the *multiple genome strategy #2*.

**Figure 3:**
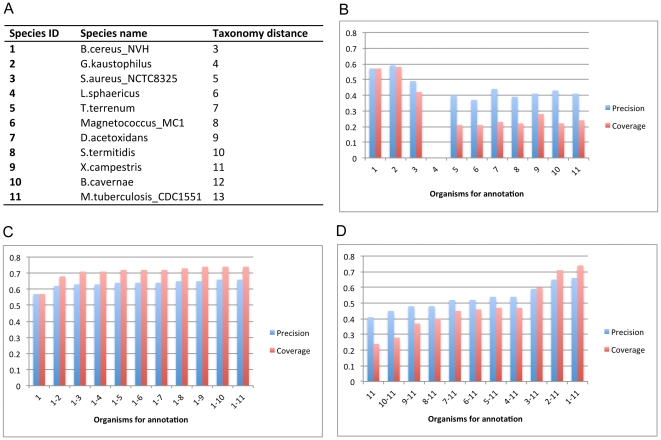
SEAS-based re-annotation of *B. subtilis* pathways using 11 reference genomes. (A) Taxonomic distance between reference genomes and *B. subtilis*. The first column represents the reference genomes, which are used in the *x*-axis in (B)–(D); (B) Re-annotation of *B. subtilis* pathways using the *single genome strategy*; (C) Re-annotation of *B. subtilis* pathways using the *multiple genome strategy #1*; (D) Re-annotation of *B.subtilis* pathways using the *multiple genome strategy #2*. *L. sphaericus* is very low in panel B at position 4 on the *x*-axis as it has no pathway annotation information.

The first two strategies have been well evaluated in the original papers on SEED [24], RAST [26] and P-MAP [27] so we focus on the assessment of the third strategy. Specifically, we will re-annotate the pathways of *E. coli* and *B. subtilis* (already in SEED) based on SEED pathways encoded by other genomes (as references). The annotation is quite time-consuming if all genomes in SEED are used as references, but the coverage could be low if only one is used considering the reference genome may not be evolutionarily close enough to contribute useful annotation templates. To balance the annotation performance and coverage, our idea is to combine some reprehensive genomes for each group of reference genomes having similar evolutional distances to the query genome. To assess this idea, we have evaluated different combinations of reference genomes in an iterative manner (Figure 2 and 3) based on the taxonomic distance, defined as the number of nodes in the path from the query organism to its closest common ancestor with its reference organism in the taxonomy tree defined in the KEGG Genome database (see Figure 2A and 3A). Based on the taxonomic distance, we have designed the following three strategies: the *single genome strategy*, which selects only one reference genome from SEED every time, but with different distance each time (see Figure 2B and 3B); *multiple genome strategy #1*, which starts with a genome in SEED having the smallest taxonomic distance to the query genome and iteratively adds the next closest genome each time until K genomes have been selected for a user selected K>0 (see Figure 2C and 3C); and *multiple genome strategy* #2, which starts from the farthest genome in SEED to the query genome and iteratively adds the next farthest genome each time until K genomes have been selected, trying to cover the best studied genomes as references, which could be close or distant. We compared the SEAS-based re-annotation results against the original pathway annotation of the two organisms in SEED using the following measures:
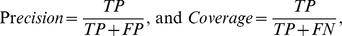
where TP (true positive) is the number of the genes for which the SEAS-based annotation is the same as the original SEED annotation, FP (false positive) is the number of the genes for which the SEAS-based annotation is different from the original SEED annotation, and FN (false negative) is the number of genes in the genome with SEED annotations but not SEAS annotations.

We observed from Figures 2 and 3 that (i) more closely related genomes generally provide more information for pathway annotation as expected (Figure 3B) although exceptions may exist such as *S. enterica* provides more information than *Y. pestis* for annotation of *E. coli* pathways possibly because *S. enterica* (55% of 4,737 genes are annotated) has more annotated genes than *Y. pestis* (51% of 5,125 genes are annotated) (Figure 2B); (ii) multiple genomes always provide more pathway-annotation information than individual genomes, also as expected; (iii) multiple genome strategy # 1 generally gives rise to more information for pathway annotation than multiple genome strategy # 2 (Figure 2C, 2D, 3C and 3D); and (iv) multiple genomes, when used in conjunction with their taxonomic distance information, have the best pathway annotation performance, achieving 55% precision and 90% coverage for *E. coli* and 66% precision and 74% coverage for *B. subtilis* For this case, 10% of original annotations for *E. coli* and 26% for *B. subtilis* are missed by SEAS (see Table S1). The missing subsystems include arabinose utilization, DNA replication, synthesis of aromatic compounds, DNA repairs for *E. coli*, and transporter, pyridoxine regulon, and spore coat, DNA repair for *B. subtilis*. Our explanation is that these subsystems do not have annotated equivalent subsystems in the reference genomes. Overall, homology search against multiple reference genomes with a wide range of different taxonomic distances generally give rise to generally good pathway annotation and can partially overcome the issue that homology search against one reference genome often leads to mapping to paralogs rather than orthologs.

### Pathway enrichment with statistic test

We have employed four statistic methods for pathway enrichment analysis, and a user can choose one as we have done with the KOBAS software [25], each of which tests whether a given gene set overlaps with a specific pathway substantially more considerably than by chance. Specifically, the four methods are hyper-geometry test, binomial test, χ^2^ test, and Fisher exact test. The hyper-geometric test requires that the input include a subset of the background annotation. If χ^2^ test is unreliable (e.g., with expected frequencies <5), SEAS will automatically switch to Fisher's exact test. The binomial test is the fastest method when the number of sequences is large [25]. We have also implemented a correction procedure for the *false discovery rate* (FDR) using *multtest* (2.8.0) provided in the R package [28], knowing that multiple hypothesis tests (one test per pathway) in each analysis performed could result in high false positive errors (see Methods and Material).

We have evaluated our pathway enrichment analysis using a gene set of *E. coli*, consisting of 42 differentially expressed genes with fold change ≥2 or ≤0.5 in the *E. coli ackA* mutant (able to produce acetyl phosphate but not metabolize it) in comparison with the *E. coli pta-ackA* mutant (unable to produce acetyl phosphate) [18]. We used the hyper-geometric test for the enrichment analyses of the SEAS and KOBAS programs. Among the 42 genes, 22 are found in the enriched KEGG pathways and 24 in the enriched SEED pathways. Seven SEED pathways are identified to be significantly enriched by SEAS, as shown in Table 1, while three KEGG pathways are identified to be enriched by KOBAS [11] (see Table S2). Overall, the results from SEAS and KOBAS are generally consistent with the result of the original study: flagella related genes play an important role in the *E. coli pta-ackA* mutant *versus ackA* mutant [18], [29]. SEAS generally gives more detailed information than KOBAS-based pathway annotation due to the nature of the SEED pathways, as in the case of type 1 pili mannose sensitive fimbriae, named as a secretion system by KEGG. Compared to KOBAS, SEAS identified three enriched pathways that KOBAS did not identify, namely colanic acid biosynthesis associated with cell adhesion [30] and lysozyme inhibitors associated with cell wall synthesis [31] without missing any significant KEGG pathways. Notably, SEAS identified b1922 as the σ^28^ factor, a minor sigma factor responsible for initiation of transcription at a number of genes involved in motility [32], while KOBAS annotates it as motility proteins and RNA polymerase, which suggests that the mutation of *pta* and *ackA* affect the activity of σ^28^ factor and thus regulates the expression of the genes related with flagellum and flagellar motility (see Table S2).

**Table 1:** Comparison between pathway enrichment analyses by KEGG- and SEED-based predictions.

KEGG pathway	FDR	SEED subsystem	FDR
Bacterial motility proteins	0	Flagellum	0
Flagellar assembly	1.0×10^−14^	Type 1 pili, mannose sensitive fimbriae	4.7×10^−8^
Secretion system	9.4×10^−3^	Flagellar motility	3.2×10^−3^
		Flagellum in Camphlobacter	3.2×10^−3^
		Bacterial chemotaxis	4.6×10^−2^
		Lysozyme inhibitors	4.6×10^−2^
		Colanic acid biosynthesis	4.6×10^−2^

FDR (false discovery rate) is a correction for high false positive errors when doing multiple hypothesis testing.

We have also compared the pathway annotation performance by the two programs on a newly sequenced genome, *N. profundicola* [33] using *E. coli* pathways in KEGG and SEED as references, respectively (using FDR≤0.05 as cutoffs). 14 out of 147 (covering 1,053 genes) KEGG pathways are enriched for *N. profundicola* and 46 out of 225 (covering 856 genes) SEED pathways are enriched, as shown in Table S3. We noted that the pathways related to ribosome, tRNA biosynthesis, transcription factor, ABC transporter, cell motility, flagella, are enriched in both KEGG and SEED. Overall, SEAS identified 31 significant pathways that the KOBAS did not identify, including folate biosynthesis, fatty acid biosynthesis, chorismate synthesis, selenocysteine metabolism, DNA repair, biotin synsthesis, histidine biosynthesis, riboflavin to FAD, purine biosynthesis, which is consistent with the conclusions in the paper [33]; while it missed six significant pathways identified by KOBAS (see Table S3). Overall SEAS and KOBAS are clearly complementary to each other as expected based on the complementary nature of their underlying pathway databases.

### Software design and implementation

The SEAS system consists of two main steps: pathway annotation and enrichment analysis, each of which can be run through a command-line, ***annotate*** and ***pathfind***, respectively. The program is implemented using the Mono cross platform (http://www.mono-project.com), open source.NET development platform, which can run on Windows, Linux and Mac OS X. All the programs are well documented, which can be quickly accessed by the ‘*-h*’ option. SEAS is released under the GNU General Public License (GPL), and the program along with related data are freely available at http://csbl.bmb.uga.edu/~xizeng/research/seas/.

SEAS runs very fast for ID-mapping based pathway annotation and pathway enrichment analysis; the only slow step of the system is the BLAST search, which takes about 1.5 hours with a single reference genome and 8.5 hours with 10 reference genomes for pathway (re)annotation of *E. coli* on a Linux workstation (6 CPUs and 8G memory). To support large-scale pathway annotation, SEAS also accepts outputs from BLAST using the (*-i blastout*) option, making the program very fast if the BLAST results are done in advance.

If the user has a list of protein sequences (fasta format), a typical session of pathway enrichment analysis is as follows:

1. Pathway annotation of the given list of proteins: seas.exe annotate –b blastp –i fasta –o “Escherichia coli,Bacillus subtilis” –f example.fasta>example.annotations, where *-b* specifies the BLAST program (blastp for protein sequence and blastx for DNA sequences), *-i* for the input format, *-o* for reference genome(s), *-f* for the input and “*>example.annotations*” specifies the output.
2. Pathway enrichment analysis with the whole *E. coli* genome as background: seas.exe pathfind –m hyper -1 example.ann -2 “Escherichia coli” >example.pathways, where -m specifies statistical method (*hyper* for hyper-geometric test, *binom* for binomial test, *chisq* for Chi Square test and *fisher* for Fisher Exact test), -1 for sample annotation file from the above step and -2 specifies background annotation file, with built-in whole genome by species name or from the above step.

### Conclusion

We have developed a new pathway enrichment analysis system, SEAS, for prokaryotes, which maps a given set of genes to SEED pathways along with a statistical significance assessment. Our evaluation result showed that SEAS-based pathway annotations tend to provide more reliable pathway predictions with slightly smaller coverage compared to a KEGG-based pathway enrichment tool KOBAS, hence it provides a new pathway enrichment tool complementary to KOBAS. We anticipate that the performance by SEAS will continue to improve as the coverage of SEED pathways continues to increase rapidly. As the only available tool specifically designed for SEED pathway enrichment analysis in the public domain, we believe that SEAS will add to the value of the SEED database, which is now being widely used by bacteriologists.

## Materials and Methods

### Data

The genome sequences and relevant annotations were downloaded from ftp://ftp.ncbi.nih.gov/genomes/Bacteria on 12/30/2010. The SEED database was downloaded from ftp://ftp.theseed.org/genomes/SEED on 12/30/2010. The KEGG database was downloaded from ftp://ftp.genome.jp/pub/kegg/genes/organisms on 12/30/2010.

### Pathway mapping from multiple reference genomes

When the query genes are not in SEED but have the sequence information, SEAS can annotate them by using the RAST server [26] or the P-Map program [27] when the whole genome is available; otherwise, SEAS annotates them based on sequence-similarity homology search against multiple reference genomes already in SEED. Specifically, SEAS does sequence similarity search for each query gene against the reference genome(s) using NCBI BLAST (blastp for protein and blastx for DNA), and selects the best hit as its mapped orthologous gene if (i) its BLAST *E-value*≤10^−5^; (ii) its *E-value* ranks among the top five hits (*Rank*≤5); and (iii) the gene has pathway information in SEED. If the user specifies multiple reference genomes, SEAS merges them into a single “genome” using the NCBI BLAST program and then applies the aforementioned algorithm for the subsequent pathway annotation. We have implemented a Ruby (http://ruby-lang.org) script to help select multiple reference genomes that are diverse in taxonomic distances. The script selects the organism out of those with the same taxonomic distance that has the most similar number of genes to that of the query genome. Currently the default value for the number of multiple reference genomes is set to be ten to ensure our aforementioned re-annotation result on the two genomes have the best precision and coverage (see Figure 2C and 3C), which can be changed by the user. The script can be freely downloaded from http://csbl.bmb.uga.edu/~xizeng/research/seas/.

### Enrichment analysis with statistic test

The statistic test methods are implemented as a separate R (http://www.r-project.org) script that is easy to extend with new methods and that to do enrichment analysis with other pathway databases outside of SEAS. The script is integrated seamlessly into the SEAS program.

## Supporting Information

Table S1 Missing annotations of E. coli and B. subtilis.
(XLS)

Table S2 Significant pathways in two E. coli mutants.
(XLS)

Table S3 Comparison of pathway enrichment analysis on the genome of *N. profundicola*.
(XLS)
